# Elicitors and cofactors of food-induced anaphylaxis

**DOI:** 10.1186/2045-7022-3-S3-P45

**Published:** 2013-07-25

**Authors:** S Hompes, L Grabenhenrich, J Grünhagen, S Dölle, M Worm

**Affiliations:** 1 Department of Dermatology and Allergy, Charité - Universitätsmedizin, Berlin, Germany; 2 Institute for Social Medicine, Epidemiology and Health Economics, Charité - Universitätsmedizin, Berlin, Germany

## Background

Food-induced anaphylaxis (FIA) is increasing and several co-factors or augmentation factors can influence the elicitation of FIA. The aim of this analysis was to investigate the role of co-factors in patients with FIA and whether double-blind, placebo-controlled food challenges (DBPCFC) can contribute to the identification of the trigger.

## Methods

100 patients with suspected FIA underwent DBPCFCs with the suspected triggers. If medical history revealed a possible influence of co-factors like physical exercise, alcohol, additives and acetylsalicylic acid (ASA), these were included into the DBPCFC protocol.

## Results

The trigger of anaphylaxis was identified in 45 out of 100 patients with suspected FIA. In 15 out of these 45 patients the identification of the trigger was achieved by the implementation of co-factors into the DBPCFCs. Food additives (n=8) followed by physical exercise (n=6), ASA (n=4) and alcohol (n=3) were identified most commonly as cofactors. The most common food allergens were celery (n=7) soy, lupine and wheat (n=4 each). In 10 patients more than one co-factor and/or more than one food allergen was required to elicit a positive reaction. The symptoms during the food challenges were weaker than those reported from the medical history.

## Conclusion

In 45 % of the patients the cause and circumstances of their reaction were identified. Although, the underlying mechanism of cofactors is not yet understood, the implementation of co-factors into the challenge protocol seems to be a worthwhile tool to increase the identification rate of elicitors in adult food anaphylactic patients.

## Disclosure of interest

None declared.

